# How the influence of cingulate-lingual interactions on event segmentation changes from early to late adolescence

**DOI:** 10.1038/s41598-026-46182-w

**Published:** 2026-04-02

**Authors:** Astrid Prochnow, Xianzhen Zhou, Foroogh Ghorbani, Veit Roessner, Bernhard Hommel, Christian Beste

**Affiliations:** 1 Cognitive Neurophysiology, Department of Child and Adolescent Psychiatry, Faculty of Medicine, TU Dresden, Schubertstrasse 42, 03107 Dresden, Germany; 2 German Center for Child and Adolescent Health (DZKJ), Partner Site Leipzig/Dresden, Dresden, Germany; 3 School of Psychology, Shandong Normal University, Jinan, China

**Keywords:** EEG, Beta frequency band, Brain connectivity, Beamforming, Event perception, Neuroscience, Psychology, Psychology

## Abstract

Event segmentation, the process of dividing continuous experience into discrete events, plays a critical role in making sense of, and navigating one’s environment. This process is likely subject to maturational changes during adolescence, but the modulation of underlying neural processes is contentious. To gain novel insights into the neurophysiological development of event segmentation, we examined *N* = 72 adolescents (13.8 ± 1.7 years, 32 males) using EEG methods and focused on modulations of theta, alpha and beta band activity. Moreover, we examined how directed connectivity between functional neuroanatomical structures associated with beta band activity across age. Connectivity from the cingulate to the lingual gyrus within the beta frequency band were significantly related to the likelihood of event segmentation, as shown by regression and moderation analyses, whereas theta and alpha band activity yielded no significant effects. The moderation analysis indicated that in older adolescents, reduced connectivity from the cingulate to the lingual gyrus likely promotes a balance between using event schemata and perceptual input when forming and updating working event models. This pattern across adolescence suggests that as the brain matures, the balanced consideration of both perceptual inputs and established episodic traces becomes increasingly relevant in the context of event segmentation. This likely enhances the efficiency of event segmentation, thereby improving the ability to organize environmental information during adolescence.

**Supplementary Information:**

The online version contains supplementary material available at 10.1038/s41598-026-46182-w.

## Introduction

Cognitive functions undergo substantial changes throughout development, with adolescence marking a particularly critical period for these transformations^1,2^. While some cognitive functions are already well-developed during this phase, others reach their full potential only by the end of adolescence^3–5^. One cognitive function that shows notable differences between adolescence and young adulthood is event segmentation^6^. Event segmentation describes how the continuous stream of information from the environment is partitioned into meaningful units to structure incoming information^7,8^. Event Segmentation Theory provides a framework for understanding the sub-processes involved in this partitioning^7,8^. Within this theoretical framework, the central element is the ‘working event model’, which represents the currently ongoing situation. This working event model is constructed using two primary sources: perceived information from the environment and information from long-term memory about how similar situations typically evolve (known as ‘event schemata’). The working event model is then used to make predictions about the near future, i.e., the next things expected to happen in the current situation. An error detection mechanism compares these predictions with the actual perceptual input. When the deviation between prediction and perception becomes too large, the current working event model is updated, resulting in the setting of an event boundary. Intriguingly, recent findings established that a prediction error is not necessary for the setting of an event boundary, but that contextual transitions can drive event segmentation as well^9–11^.

Previous research has shown that adolescents are less likely than adults to set event boundaries, probably due to a lesser extent of available event schemata^6^. Additionally, differences in working memory processes that are relevant to the creation and maintenance of the working event model, as well as in the functioning of error detection mechanisms might contribute to these discrepancies, as both continue to develop during adolescence^12,13^. Adolescents are more lenient in detecting errors as compared to young adults^13^, suggesting potential differences in cognitive processing mechanisms between age groups. Critically, existing studies primarily highlight general differences between adolescents and adults without focusing on the detailed development of cognitive processes during adolescence^3,4,6,12,13^. Given the tremendous cognitive changes occurring throughout adolescence, it is crucial to investigate this developmental trajectory in more detail.

Importantly, the substantial developmental changes during adolescence are also observable at the neurophysiological level, including variations in oscillatory activity^14,15^ and the dynamics of brain networks^4,16^. These changes are significant as oscillatory dynamics are central to information coding^17–19^. Of particular importance for our purposes, alpha, beta, and theta frequency bands undergo maturational processes during adolescence. In the context of event segmentation they are suggested to correspond to different segmentation sub-processes as conceptualized by event segmentation theory^20^. The beta frequency band appears to be associated with the maintenance and updating of the working event model, while the alpha frequency band is thought to be related to accessing event schemata^20^. The theta frequency band, known for its role in signaling surprise and error detection^21–23^, is thought to reflect the error detection mechanism that compares predictions from the working event model with the actual occurrences in a situation^20^. Moreover, theta band activity has been shown to be involved in event segmentation, likely due to its role for memory formation and retrieval^24,25^. Given that there are notable differences in the oscillatory power of these frequency bands between adolescents and adults, maturational processes throughout adolescence likely affect all three frequency bands. However, since different brain regions and their associated cognitive functions develop at varying time scales during adolescence^1–3^, it is plausible that the three frequency bands and their associated sub-processes mature at different speeds during this period. Moreover, connectivity between brain regions—and thus the communication within the brain—also changes throughout adolescence^1,26–29^ and should thus be taken into account when investigating the developmental changes in event segmentation.

The current study therefore aims to investigate the contribution of power in alpha, beta and theta frequency bands, as well as the connectivity between relevant brain regions within these frequency bands, to event segmentation behavior. In line with previous work^20,30,31^, event segmentation will be examined using a segmentation task in which participants evaluate a movie (see Materials and Methods for details). Segmentation is quantified as the likelihood of placing an event boundary based on the information available in the movie^31^. While previous research on brain connectivity has relied on sensor-level metrics such as small-world networks^32,33^ or source-level approaches focusing on either solely linear or solely non-linear relationships, this study employs a connectivity analysis that estimates linear and non-linear interactions among specific loci of activity within these frequency bands at the same time, which is of particular relevance as is has been shown that neurophysiological signals contain a substantial proportion of nonlinear contributions^34,35^. Moreover, by applying this method, we build on a previous study by Ghorbani et al.^6^ who identified medial frontal and temporo-occipital regions as being involved in event segmentation in adolescents across all three frequency bands, suggesting a similar pattern of brain region involvement is expected in the current study. We hypothesize that the relationship between neurophysiological signals in these brain regions and event segmentation behavior will depend on age, with the three frequency bands and involved brain regions likely following different developmental trajectories. Additionally, the directed connectivity between the obtained brain regions of interest in frequency bands important for event segmentation behavior is expected to influence event segmentation, with this relationship potentially varying by age throughout adolescence.

## Methods

### Sample

Data of *N* = 90 healthy controls aged between 10 and 16 years were collected. *N* = 2 participants had to be excluded due to psychiatric diagnosis according to the parent’s report in a questionnaire, *N* = 2 participants were excluded due to technical errors in the script for the behavioral analysis, and *N* = 9 participants had to be excluded due to problems with EEG recording and analysis (recording started after beginning of the task, insufficient quality, error in segmentation). Of the remaining *N* = 77 participants, *N* = 4 were excluded as outliers regarding their number of responses in the task (more than three scaled median absolute deviation away from the median). Thus, the final sample consisted of *N* = 73 participants (13.7 ± 1.7 years, range 10–16 years, 33 males). However, after the nCREANN analysis (see below), *N* = 1 additional subject had to be excluded due to poor performance of the nCREANN algorithm on the participant’s data set, thus the sample for analyses using values from nCREANN consisted of *N* = 72 participants (13.8 ± 1.7 years, range 10–16 years, 32 males). Participants were recruited via an in-house database and advertisements. Written informed consent was given by all of the participants and their legal guardians before any study procedure was applied. The study was approved by the local ethics committee of the Medical Faculty of the TU Dresden.

### Ethical considerations

Written informed consent was given by all of the participants and their legal guardians before any study procedure was applied. The study was approved by the local ethics committee of the Medical Faculty of the TU Dresden (IRB00001473) and was conducted in accordance with the Declaration of Helsinki.

### Task

Participants watched the short film “The Red Balloon”^36^ while performing an event segmentation task, comparable to other work by our group^6,20,37,38^ and based on previous work on event segmentation^31,39–42^. For this task, they were instructed to press the space key with their right index finger whenever they perceived that something had ended and something new was about to begin. There were no specific guidelines regarding the length or content of these meaningful segments. The film was selected based on prior research because it features a linear timeline, minimal spoken language, and continuous, identifiable situational changes^8,31,40^. Before watching “The Red Balloon”, participants viewed a short film of a few minutes showing a man preparing a room for a party^43,44^ to familiarize themselves with the task and ensure they understood the instructions. “The Red Balloon” (total duration of about 34 min) was then presented to participants using the software Presentation (RRID:SCR_002521) in three clips, each lasting about 10 min, with breaks of self-determined length between them. We then applied the situational change coding established by Zacks et al.^31^ to count the number of changes within each 2-s time bin of the movie. These changes could be, for instance, changes in the agents on screen, changes in interactions between agents, changes in camera perspective, changes in location of agents, or changes on object use by agents. In this way, the movie was divided into 978 intervals, of which 512 intervals did not contain any situational changes, 276 intervals contained one situational change, 104 intervals contained two situational changes, 46 intervals contained three situational changes, 30 intervals contained four situational changes, and 10 intervals contained five or more situational changes. To operationalize event segmentation, the key presses given by the participants were recorded as behavioral outcome of the task. Notably, there is no objective template for when an event boundary should be placed, so performance in this task cannot be evaluated against a definitive standard.

### EEG recording and preprocessing

The EEG signals were measured using Ag/AgCl electrodes placed in an equidistant layout, with the reference electrode at Fpz and the ground electrode positioned at θ = 58/ϕ = 78. The signals were recorded via 60 electrodes using a BrainAmp amplifier (Brain Products Inc.) while participants performed the event segmentation task. Details about the task stimuli (i.e., start and end of movie clips) and key presses were logged along with the EEG data. During electrode preparation, it was ensured that impedances were below 5 kΩ. For preprocessing, the data were initially down-sampled from 500 to 300 Hz. The subsequent steps involved using the “Automagic” pipeline^45^ and EEGLAB^46^ (RRID:SCR_007292) with MATLAB (The MathWorks Corp; http://www.mathworks.com/products/matlab/; version R2021b; RRID:SCR_001622). First, flat channels were discarded and the EEG data were re-referenced to an average reference. Next, the PREP pipeline^47^ and EEGLAB’s “clean rawdata()” pipeline were used. The PREP pipeline removed line noise (50 Hz) using a multitaper algorithm, discarded channels with high variance (threshold at 50 µV), and established a robust average reference. In the “clean rawdata()” pipeline, a high-pass filter at 0.5 Hz (FIR; order 1286; stop-band attenuation -80 dB; transition band 0.25–0.75 Hz) was applied to detrend the data, and flat, noisy, and outlier channels were removed. The Artifact Subspace Reconstruction (ASR; burst criterion: 15^48^) was then used to reconstruct segments with abnormally high power (> 15 standard deviations from calibration data), with unrepairable segments removed. A low-pass filter at 40 Hz (sinc FIR filter; order: 86^49^) was subsequently applied. EOG artefacts were removed using a subtraction method^50^, and remaining artefacts (e.g., muscle, heart, and residual eye artefacts) were identified and eliminated through independent component analysis (ICA) using the multiple artifact rejection algorithm (MARA^51,52^). Finally, removed channels were interpolated using a spherical method, resulting in a set of 60 electrodes for subsequent analyses for each participant. If channel locations have not been stored correctly, 20 or more channels have been interpolated, or the beginning of the movie has not been recorded, the participant was excluded.

Following these preprocessing steps, the neurophysiological data were segmented using the FieldTrip toolbox^53^ (https://www.fieldtriptoolbox.org; version fieldtrip-20230602; RRID:SCR_004849) based on the responses given by the participant. Segments were locked to the time point of the response, ranging from − 1 s to 1 s relative to the time point of the response. If responses were less than 2 s apart, the later response was discarded to avoid having more than one response in a segment of interest. In this way, the segments were substantially longer than the time window typically assumed for response preparation, as reflected, for example, by the lateralized readiness potential^20^.

### Time–frequency decomposition and cluster-based permutation testing

In order to identify the frequency bands that were modulated around event boundaries, we compared activity around participant-defined boundaries with activity in non-boundary intervals. Based on the results of this sensor level analysis, the frequency bands of interest for source level analysis are chosen. Following previous work^6,20,38^, virtual markers were created using the FieldTrip toolbox^53^ as follows: (1) Neurophysiological data were divided into 2-s bins, labeled as Boundary Intervals (BI) if they contained a response by the participant, or No-Boundary Intervals (NBI) if they did not. (2) For each BI, the precise response time within the bin was determined, and a − 2 to 2 s window around the marker was checked for additional responses or data issues. (3) Since NBIs outnumbered BIs, a matching number of NBIs were randomly selected per participant, and virtual markers were placed at the same relative time within each NBI as in its paired BI. These markers were then validated as in step 2. If any virtual markers were invalid, the process was repeated until all were valid. After determining all marker positions, the data were segmented from − 2 to 2 s relative to the marker. This segmentation into longer segments was necessary to avoid edge effects in the time window of interest from − 1 to 1 s around the markers. Although this control condition does not contain a response, as the condition of interest does, other control conditions would introduce additional confounds, such as different decision processes when using a free key-pressing task or reduced attentional focus if participants were watching the movie passively^20^.

Time–frequency analysis was conducted using a wavelet-based approach in the FieldTrip toolbox to compute power across the frequency range of 3–30 Hz. A Morlet wavelet with a width parameter of 5 was used. To achieve the desired frequency resolution, zero-padding was utilized, where the length of the padding was set to the ceiling of the trial length in samples (2 s, i.e., 600 samples) divided by the sampling rate of 300 Hz. Power was estimated at all available time points in the segmented data. Due to the chosen time window, which was selected to focus on processes around the event boundaries, it was not possible to reliably resolve frequencies below 3 Hz, such as slow theta or delta waves.

Statistical comparisons between conditions were conducted using non-parametric permutation tests in the FieldTrip toolbox. The analysis was performed in each frequency band of interest (theta frequency band: 4–7 Hz; alpha frequency band: 8–12 Hz; beta frequency band: 15–30 Hz) averaged across the time window from -1 to 1 s. A cluster-based permutation test was applied with 1000 randomizations and a significance threshold of *p* < 0.05. To control for multiple comparisons, cluster correction was applied with a cluster threshold of *p* < 0.05. The statistic used for comparisons was the dependent-samples t-test. A minimum of 2 neighboring channels was required for cluster formation. The statistical analysis was performed with averaging over both frequency and time.

### Beamforming analysis

Based on previous work by Ghorbani et al.^6^, we analyzed the sources of the activity in intervals centering around the responses in BI intervals. Focusing on only one condition instead of contrasts was necessary to estimate the functional connectivity in the subsequent connectivity analysis (see below^34^) and further brings the advantage that motor-related processes can be assumed to be similar across all subjects. Beamforming analysis, an established approach for EEG source localization^54,55^, was conducted for these segments, with the time of interest for the beamforming being set to − 0.5 s to 0.5 s to focus the analysis on the time point of segmentation behavior. Prior to the beamforming procedure, electrode Fpz was interpolated and data were re-referenced again to a common average reference. To analyze source activity in the specified frequency bands, we utilized dynamic imaging of coherent sources (DICS) beamforming^56^. For DICS beamforming, we created common spatial filters for each individual based on the cross-frequency spectra obtained from Fast Fourier Transformation (FFT) of the averaged theta (4–7 Hz), alpha (8–12 Hz) and beta (15–30 Hz) band data. The DICS beamformer projected activity locations onto a grid with 0.5 cm spacing, using the FieldTrip toolbox’s forward model template aligned with the standard Montreal Neurological Institute (MNI) space as the source space as individual MRIs were not recorded. To account for increased noise towards the center of the head, we calculated the neural activity index (NAI) by dividing source estimates by local noise estimates for each voxel^57^. We then used the Density-Based Spatial Clustering of Applications with Noise (DBSCAN) algorithm to identify clusters of pronounced activity within functional neuroanatomical regions in the frequency bands of interest^58,59^. A threshold at the top 1% of the power distribution within regions defined by the automatic anatomical labeling (AAL) atlas^60^ was set to focus the analysis on voxels with the highest activity in each frequency band. DBSCAN identified neighboring voxels with an epsilon value set to 1.5*the edge length and a minimum cluster size of five voxels. We validated the DBSCAN results through manual inspection, considering cluster size and corresponding AAL atlas labels. The methodological details and results of a similar procedure additionally conducted for the contrasts of BI and NBI can be found in the Supplementary Material (Suppl. Analysis 1, Suppl. Fig. 1).

To estimate the directed linear and nonlinear connectivity between clusters obtained by the DBSCAN algorithm, it is not only necessary to have the localization of activity in the brain, but its time course as well. To this end, linearly constrained minimum-variance (LCMV) beamforming^57^ was applied to the pre-processed and segmented EEG data. Thereby, the clusters obtained using the DBSCAN algorithm were used as regions of interest. This method enables the reconstruction of time series data from source regions based on the recorded scalp activity. The LCMV beamformer achieves this by applying a spatial filter, calculated from the covariance matrix of the time-locked averaged data for each cluster, to the EEG data. For the beamforming computation, a lead field with a 0.5 cm grid resolution was generated using the Montreal Neurological Institute (MNI) coordinate system provided by the FieldTrip toolbox. We computed the LCMV beamformer for each subject using data from all electrodes within the time frame from -0.5 to 0.5 s relative to the time of a response, resulting in time courses of the activity in the time domain. Based on the results of the regression and moderation analyses (see below), only time courses of beta band activity for each source were computed by applying a time–frequency analysis on the voxel time series data, covering a frequency range of 15 to 30 Hz in 1 Hz steps, using a Morlet wavelet with a parameter of 5. Beta power was averaged across the 15 to 30 Hz range.

### Connectivity analysis

The assessment of directed connectivity was performed using the nCREANN (nonlinear Causal Relationship Estimation by Artificial Neural Network) method, which utilizes an artificial neural network to estimate effective connectivity between multiple brain regions. Unlike traditional linear methods that primarily focus on linear Multivariate Autoregressive (MVAR) models, nCREANN accounts for both linear and nonlinear dynamics separately in the flow of information across cortical regions. By employing MVAR models, which describe how a signal is influenced by its past values and those of other signals, this method enables the determination of the direction of connectivity. This approach is essential for comprehending the complex and nonlinear nature of information transfer among cortical regions, as linear methods may oversimplify the intricate dynamics of brain function by overlooking nonlinear aspects. The nCREANN algorithm has previously been evaluated using simulated nonlinear MVAR models with known ground truth connectivity structures, demonstrating accurate recovery of linear and nonlinear causal interactions^34^.

As there is substantial evidence that understanding neuro-dynamics on a macroscale, involving brain regions engaged in various functions, necessitates consideration of both linear and nonlinear principles, the nCREANN method distinguishes between linear and nonlinear causal relationships. In a nonlinear MVAR model, the current samples of brain regions are generated based on the interactions of previous regions’ activities, as described by the equation:

1 $${\mathrm{x}}\left( n \right) = f\left( {{\mathrm{x}}_{p} } \right) + {\upsigma }\left( n \right)$$

Here, $\mathrm{x}\left(n\right)$ represents the vector of M time series (regions of interest) at the current time point, while ${\mathrm{x}}_{p}={\left[{x}_{1}\left(n-1\right),{x}_{2}\left(n-1\right), \cdots ,{x}_{M}\left(n-p\right) \right]}^{\mathrm{T}}$ denotes the vector of their *p* previous samples. The term $\upsigma \left(n\right)= {\left[{\sigma }_{1}, {\sigma }_{2}, \dots , {\sigma }_{M} \right]}^{T}$ represents the model residual. This model is implemented through a single-hidden-layer feed-forward network. During training, the network is supplied with past samples of the source signal to predict the current samples, which serve as the network’s output. The nonlinear activation functions for the hidden neurons and linear functions at the output layer are encapsulated in $f\left(.\right)$, a nonlinear function that expresses how the *p* previous samples affect the present values. This results in a nonlinear MVAR model, embedding information about both linear and nonlinear interactions in the network’s parameters.

To discern effective connectivity from the input to the output of the network, the Taylor expansion of the activation function of the hidden neurons is used to separate the linear and nonlinear components of $f\left(.\right)$:

2 $$f = f^{Lin} + f^{NonLin}$$

The Taylor expansion is a mathematical technique that approximates a function through the summation of linear and nonlinear terms. The linear terms are first-degree polynomial terms, while the nonlinear terms involve higher-degree polynomial terms. In the nCREANN method, ${f}^{Lin}$ is calculated based on the input–output mapping that solely represents the linear interactions among signals, while the nonlinear counterpart ${f}^{NonLin}$ is inferred from the estimation error ratio. Therefore, linear effective connectivity from region *i* to region *j* $\left({LC}_{i\to j}\right)$ is extracted from ${f}^{Lin}$ by multiplying the connection weights of the network with the scaling parameters of the hidden neurons. Conversely, nonlinear effective connectivity $\left({NC}_{i\to j}\right)$ ​is defined as the ratio of the network’s estimation errors (the discrepancy between original values and predicted values):

3 $$NC_{i \to j} = \ln \left( {\frac{{\left( {\epsilon_{j} } \right)_{{x_{i} \_Lin}}^{2} }}{{\left( {\epsilon_{j} } \right)^{2} }}} \right)$$

In this equation, the denominator indicates the estimation error when all input signals collectively exert both linear and nonlinear influences on *x*_*j*_. The numerator measures the effect on *x*_*j*_​ after removing the influence of region *x*_*i*_ from the network’s input while allowing other signals to exert both linear and nonlinear influences on *x*_*j*_. Thus, ${NC}_{i\to j}$ quantifies the degree to which *x*_*i*_ exerts a nonlinear causal effect on *x*_*j*_.

In this study, a nonlinear MVAR model was implemented using a single hidden layer feedforward network with 6 hidden neurons, trained via gradient descent error back-propagation (EBP) with momentum (α) and an adaptive learning rate (η). To promote generalization, early stopping was applied. The data was divided into training (80%), validation (10%), and testing (10%) sets using a fivefold permuted cross-validation technique. The network parameters were updated in ‘incremental’ mode (with each presentation of an input to the network), initialized randomly within the range of [− 0.5, 0.5]. To ensure effective network training, all data points from − 0.5 to 0.5 s around a response were utilized, and these intervals were concatenated to achieve a sufficient data length. The optimal model order (*p* = 9) was determined using the Akaike and Schwartz criteria. Model evaluation was conducted using the R^2^ criteria based on the testing data. R^2^, known as the coefficient of determination, serves as a measure of goodness of fit in regression problems, with values close to 1 indicating a good model fit.

### Statistical analyses

The analysis of the behavioral data was conducted for each participant using a logistic regression model with the predictor number of changes per 2-s interval (0–5) and the occurrence of a response within a 2-s interval as the outcome (0—no response; 1—response occurred). This was realized using the ‘glmfit’ function as implemented in MATLAB (RRID:SCR_001622). The distribution was assumed to be binomial, and a logit link function was defined for the relationship between predictor and outcome. The computed coefficient estimates were used for the subsequent analysis. To estimate the effect of the number of changes on the response for the whole sample, a mixed-effects logistic regression model was computed in R (RRID:SCR_001905) using the ‘glmer’ function, taking the variability between participants into account by modelling a random intercept for participants.

Finally, the coefficient estimates from the single-subject logistic regression analysis (i.e., how strongly the changes in the movie influence event segmentation response behavior), the NAI values of each identified cluster and the age of each participant were used for a moderation analysis in SPSS (RRID:SCR_016479), using the PROCESS macro (v4.0 for SPSS) by Hayes^61^ which is based on an ordinary least squares regression analysis. The NAI values were used as predictors, while age was used as moderator and the coefficient estimates from the logistic regression analysis were defined as outcome. Heteroscedasticity-consistent inference was taken into account (HC3^62^) and the Johnson-Newman technique was applied in case of significant moderation effects^63,64^. Using this technique, it is possible to identify those values on the continuum of the moderator variable for which the effect of the predictor changes its significance level^61^. Moreover, to evaluate the effects of the NAI values on the coefficient estimates independent of age, linear regression analyses were calculated in SPSS, using the NAI values as predictors and the coefficient estimate as outcome. Furthermore, as the results pointed to a crucial role of the clusters identified in the beta frequency band (see Results), regression and moderation analyses were conducted using the directed connectivity values obtained by nCREANN as predictors.

False discovery rate was controlled using the Benjamini–Hochberg procedure (q = 0.05). Separate correction families were defined for regional neural activity predictors and functional connectivity predictors, reflecting distinct neurobiological hypotheses. Within each family, correction was applied separately for main effects and age moderation effects.

## Results

### Behavioral results

**Fig. 1:**
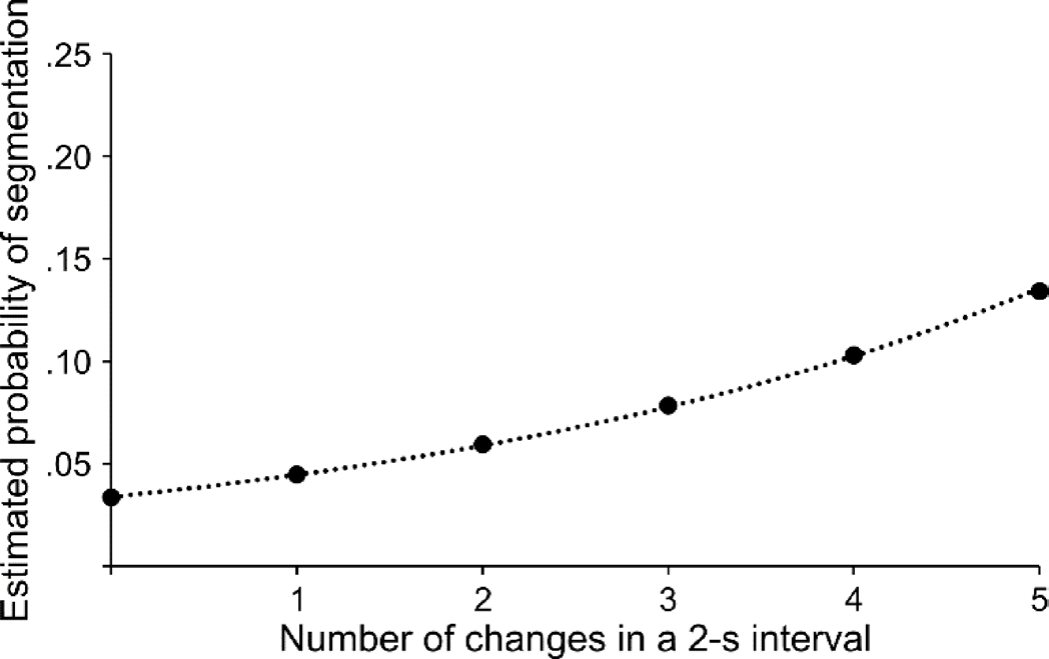
Behavioral results. The estimated probability of segmentation behavior (y-axis) is displayed as a function of the number of situational changes in a 2-s interval (x-axis).

The mixed-effects logistic regression revealed a significant intercept (− 3.36, *p* < 0.001, OR = 0.03, 95% CI 0.03–0.04) and a significant influence of the number of changes in a 2-s interval on the probability of a response (0.30, *p* < 0.001, OR = 1.35, 95% CI 1.32–1.38). Thus, with an increasing number of situational changes, also the probability of event segmentation behavior increases. This relationship between the number of changes and the probability of a response is visualized in Fig. 1.

### Results of cluster-based permutation testing

**Fig. 2:**
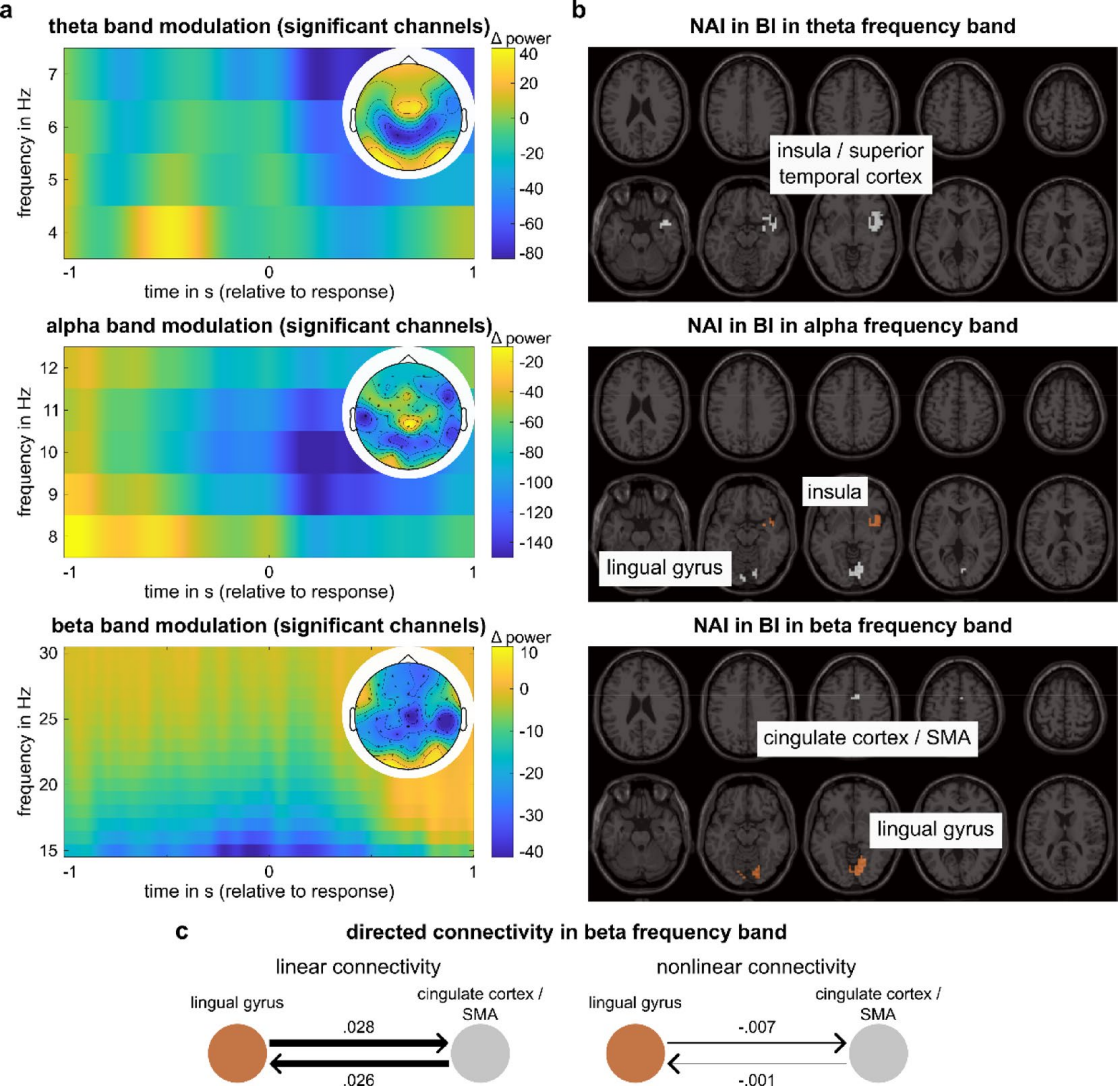
Time–frequency decomposition, DBSCAN and nCREANN results. (**a**) Illustration of the difference of the time–frequency values in Boundary minus No-Boundary intervals at sensor level averaged across significant electrodes for theta (4–7 Hz), alpha (8–12 Hz) and beta (15–30 Hz) frequency bands, along with the plots of the cluster-based permutation tests averaged across time. In the topographic cluster plots, significantly different sensors are marked with a cross (*p* < 0.050) or an asterisk (*p* < 0.010). Time point zero denotes the time of the response/virtual marker. (**b**) Clusters of voxels with the highest 1% of NAI in the respective frequency band. (**c**) Schematic illustration of the directed connectivity between clusters in the beta frequency band resulting from the nCREANN algorithm, separately for linear and nonlinear connectivity.

The modulation of oscillatory activity between BI and NBI in all three frequency bands at sensor level is illustrated in Fig. 2a. In the theta frequency band, the cluster-based permutation testing revealed a negative cluster (T_sum_ = − 23.65, *p* = 0.018) across central and parietal electrodes (CP3, P1, P3, C4, CP4, P2, PZ, C6). With respect to alpha frequency band, cluster-based permutation testing established a negative cluster (T_sum_ = − 349.16, *p* = 0.002) across all electrodes. Regarding the beta frequency band, cluster-based permutation testing showed a significant negative cluster (T_sum_ = − 331.64, *p* = 0.002) across all electrodes except electrode O9. Thus, activity in all three frequency bands was decreased in BI compared to NBI.

### Results of beamforming and subsequent regression and moderation analysis

Concerning the beamforming analysis in BI, in the theta frequency band, one right-hemispheric cluster was obtained encompassing the insula and the superior temporal pole and spanning to the superior and middle temporal lobe and the orbitofrontal cortex (including BA 13/21/38). The highest activity in the alpha frequency band in BI was found in one right-hemispheric cluster encompassing parts of the insula, the superior temporal lobe, the superior temporal pole (including BA 13), and a second cluster in the bilateral lingual and calcarine gyri (including BA 18/19). Regarding the beta frequency band in BI, one cluster with the highest activity was found in the bilateral lingual and the left calcarine gyri (including BA 18/19), and a second cluster encompassing the cingulum and the supplementary motor area (including BA 6/24). The location of the clusters is visualized in Fig. 2b.

The results of the regression analyses using the activity in the obtained clusters and the connectivity between the regions in the beta frequency band (see below), respectively, as predictors and the individually estimated coefficient of the logistic regression as outcome are listed in Table 1.

**Table 1:** Results of the regression analyses with the individually estimated coefficient of the logistic regression as outcome.

Predictor	Regression analysis outcomes				
	R^2^	F	*p*	p_corr_	Durbin–Watson
TBA insular	0.002	0.11	0.740	0.925	2.14
ABA insular	0.003	0.22	0.643	> 0.999	2.14
ABA lingual	0.002	0.13	0.725	> 0.999	2.11
BBA lingual	0.000	0.00	0.988	0.988	2.13
BBA cingulum/SMA	**0.075**	**5.72**	**0.019**	0.095	2.20
BBA linear connectivity lingual to cingulum/SMA	0.001	0.10	0.748	> 0.999	2.14
BBA linear connectivity cingulum/SMA to lingual	0.005	0.38	0.541	> 0.999	2.14
BBA nonlinear connectivity lingual to cingulum/SMA	0.000	0.03	0.871	> 0.999	2.14
BBA nonlinear connectivity cingulum/SMA to lingual	0.000	0.00	0.959	0.959	2.13

TBA, theta band activity; ABA, alpha band activity; BBA, beta band activity; SMA, supplementary motor area. P_corr_ indicates *p*-value after Benjamini–Hochberg correction. Significant values are printed in bold.

Regarding the theta band activity in the right temporal cortex, when adding age as moderator variable, there was a significant determination coefficient (R^2^ = 0.126, F(3,69) = 3.35, *p* = 0.024), but the interaction of theta band activity and age did not reach significance (ΔR^2^ = 0.005, b = 0.001, F(1,69) = 0.35, *p* = 0.554, p_corr_ = 0.693). Thus, the influence of theta band activity on the behavioral outcome was not moderated by age.

Concerning the alpha frequency band, the moderation analysis including alpha band activity in the insular cortex as predictor and age as moderator variable revealed a significant regression model (R^2^ = 0.125, F(3,69) = 3.21, *p* = 0.028), however, the interaction term was not significant (ΔR^2^ = 0.003, b = 0.001, F(1,69) = 0.25, *p* = 0.616, p_corr_ = 0.616). Similarly, adding age as a moderator to the model using alpha band activity in the lingual gyrus as a predictor explained a significant proportion of variance in the outcome measurement (R^2^ = 0.135, F(3,69) = 3.43, *p* = 0.022), but the interaction term did not reach significance (ΔR^2^ = 0.008, b = 0.002, F(1,69) = 0.41, *p* = 0.526, p_corr_ = 0.877).

Regarding the regression model using beta band activity in the lingual gyrus as a predictor, when adding age as a moderator, the determination coefficient of the model was significant (R^2^ = 0.134, F(3,69) = 3.42, *p* = 0.022). However, the interaction of beta band activity in the lingual gyrus with age did not reach significance (ΔR^2^ = 0.010, b = 0.002, F(1,69) = 0.69, *p* = 0.410, p_corr_ > 0.999). Similarly, concerning the cluster encompassing the cingulate cortex and the SMA in the beta frequency band, when adding age as a moderator variable, the determination coefficient was significant (R^2^ = 0.180, F(3,69) = 4.94, *p* = 0.004), but the estimated coefficient of the interaction of beta band activity in the cingulum and SMA with age (ΔR^2^ = 0.018, b = -0.002, F(1,69) = 2.06, *p* = 0.156, p_corr_ = 0.780) did not reach significance.

### Directed connectivity results and subsequent regression and moderation analysis

Based on the results of the regression and moderation analysis, the connectivity analysis using nCREANN was only conducted for the connectivity between the two clusters in the beta frequency band, as this was the only case in which at least marginally significant results (uncorrected) were observed. One participant had to be excluded due to a poor performance of the nCREANN algorithm on this participant’s data set and is therefore not part of the subsequent regression and moderation analyses including the connectivity measures as predictors. Apart from that, the model showed a very good fit across subjects (R^2^ = 0.98). A schematic illustration of the connectivity between the regions is presented in Fig. 2c. There was no difference between the directed linear connectivity from the lingual gyrus to the cingulate cortex/SMA (0.028 ± 0.013) and the directed linear connectivity from the cingulate cortex/SMA to the lingual gyrus (0.026 ± 0.014; t(71) = 1.33, *p* = 0.189). Further, also the difference between the directed nonlinear connectivity from the lingual gyrus to the cingulate cortex/SMA (0.007 ± 0.056) and the directed nonlinear connectivity from the cingulate cortex/SMA to the lingual gyrus (0.001 ± 0.074) was not significant (t(71) = 0.56, *p* = 0.575).

**Fig. 3:**
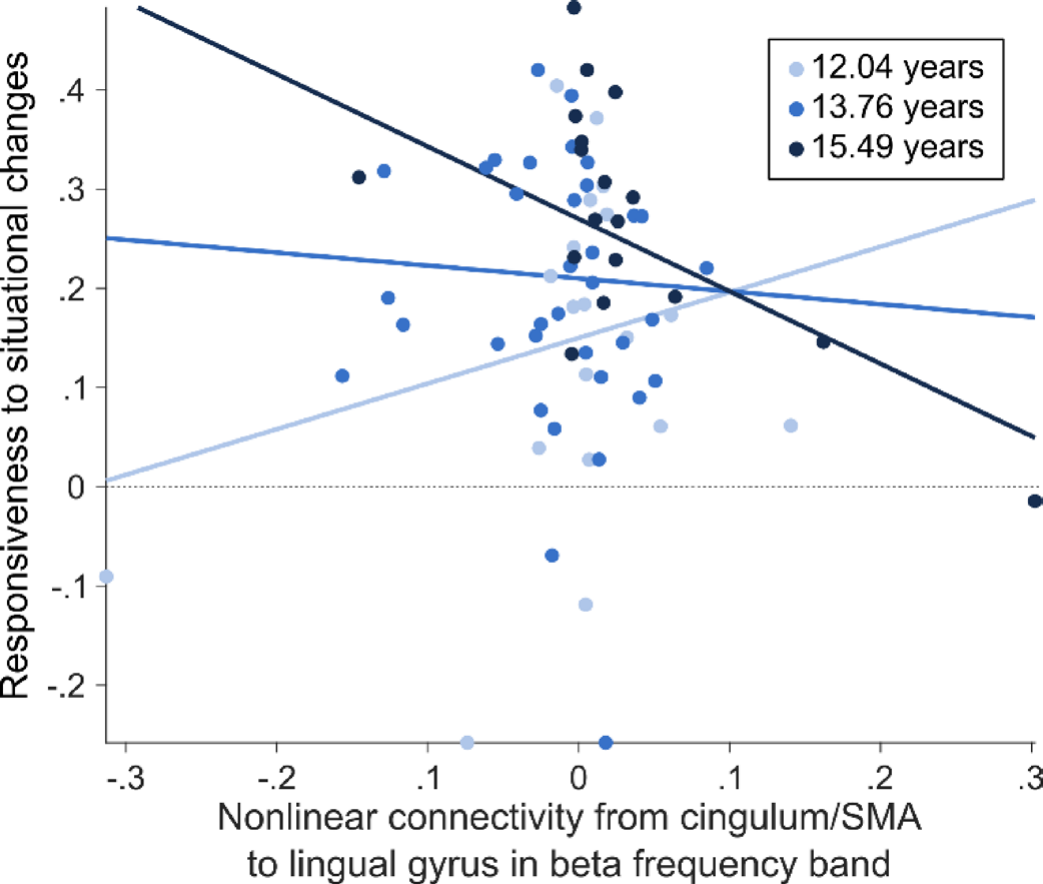
Moderation by age. The moderation by age is displayed for the responsiveness to situational changes (y-axis) as a function of the directed nonlinear connectivity from the cingulate gyrus/SMA to the lingual/calcarine gyri (x-axis). The light blue line indicates the group aged one standard deviation below the mean age, the dark blue line indicates the group aged one standard deviation above the mean age, and the medium blue line indicates the group aged within one standard deviation around the mean age. The dots indicate the values of the single subjects, with the color indicating the respective group they belong to.

The results of the regression analyses with the directed connectivity as predictor and the estimated coefficient as outcome are displayed in Table 1. With respect to the influence of the directed connectivity on event segmentation behavior moderated by age, for the linear connectivity from the lingual gyrus to the cingulate gyrus/SMA and vice versa as well as for the nonlinear connectivity from the lingual gyrus to the cingulate gyrus/SMA, the moderation analysis models revealed significant overall determination coefficients, but no significant interaction effects between predictor and moderator (linear connectivity lingual gyrus to cingulum/SMA: model: R^2^ = 0.135, F(3,68) = 3.90, *p* = 0.012, interaction: ΔR^2^ = 0.001, b = 0.161, F(1,68) = 0.06, *p* = 0.807, p_corr_ > 0.999; linear connectivity cingulum/SMA to lingual gyrus: model: R^2^ = 0.141, F(3,68) = 3.96, *p* = 0.012, interaction: ΔR^2^ = 0.001, b = -0.186, F(1,68) = 0.05, *p* = 0.827, p_corr_ = 0.827; nonlinear connectivity lingual gyrus to cingulum/SMA: model: R^2^ = 0.135, F(3,68) = 4.45, *p* = 0.007, interaction: ΔR^2^ = 0.003, b = 0.080, F(1,68) = 0.14, *p* = 0.708, p_corr_ > 0.999). Importantly, when adding age as a moderator to the regression model encompassing the nonlinear connectivity from the cingulate cortex/SMA to the lingual gyrus as predictor and the responsiveness to situational changes as outcome, the model explained a significant proportion of variance (R^2^ = 0.228, F(3,68) = 7.423, *p* < 0.001) and established a significant moderation of the effect by age (ΔR^2^ = 0.090, b = -0.346, F(1,68) = 7.78, *p* = 0.007, p_corr_ = 0.028; Fig. 3). Thus, there was a significant moderation of the relationship between the directed nonlinear connectivity from the cingulate to the lingual gyrus and the estimated coefficient of the logistic regression by age. As Cook’s distance was ≤ 0.55, it can be assumed that there were no outliers affecting the regression slopes in the three age groups (12.04 years, 13.76 years, 15.49 years; see also Fig. 3).

The Johnson-Newman output revealed that there was a significant relationship between the directed linear connectivity from the lingual to the cingulate gyrus and the estimated coefficient of the logistic regression only from the age of 14.4 years (see Table 2). Before that age, the relationship was not significant.

**Table 2:** Johnson-Newman output for the moderation analysis employing the nonlinear connectivity from cingulate gyrus and SMA to lingual gyrus as predictor, age as moderator and the individually estimated coefficient of the logistic regression as outcome.

Age (in years)	coefficient estimate	95% confidence interval–lower boundary	95% confidence interval–upper boundary	t(71)	*p*
**10.00**	1.17	− 0.08	2.42	1.86	0.067
**10.60**	0.96	− 0.15	2.07	1.73	00.089
**11.20**	0.76	− 0.22	1.73	1.55	0.125
**11.80**	0.55	− 0.29	1.38	1.31	0.194
**12.40**	0.34	− 0.36	1.04	0.97	0.336
**13.00**	0.13	− 0.44	0.71	0.46	0.647
**13.60**	− 0.08	− 0.54	0.39	− 0.32	0.747
**14.20**	− 0.28	− 0.66	0.09	− 1.49	0.140
14.42	− 0**.36**	− 0**.71**	**0.00**	− **2.00**	**0.050**
**14.80**	− 0.49	− 0.83	− 0.15	− 2.89	0.005
**15.40**	− 0.70	− 1.06	− 0.34	− 3.84	< 0.001
**16.00**	− 0.91	− 1.34	− .47	− 4.13	< 0.001

The values printed in bold font indicate the borderline of significance.

In summary, the results showed that only beta band activity in the cingulate gyrus and SMA predicted event segmentation behavior. Concerning the dependence on age, only the relationships of the nonlinear connectivity from the cingulate gyrus and SMA to the lingual gyrus with the event segmentation behavior were moderated by age. More specifically, after the age of 14.4 years, the higher the nonlinear connectivity from the cingulate gyrus/SMA to the lingual gyrus, the lower the dependence of event segmentation behavior on situational changes.

## Discussion

The current study aimed to investigate the relationship between event segmentation behavior and its neurophysiological underpinnings throughout adolescence with particular emphasis on the role of network connectivity profiles in different frequency bands. To this end, adolescents performed an event segmentation task^30,31^ while EEG was recorded concomitantly. The EEG data was analyzed using beamforming techniques and applying connectivity measurements (nCREANN^34^). The relationship between neurophysiological activity and connectivity on the one hand and event segmentation behavior on the other hand was examined concerning age during adolescence utilizing moderation analysis.

The behavioral results demonstrated the typical relationship between the number of changes in a time interval and the likelihood of exhibiting segmentation behavior: more changes increased the probability of setting an event boundary^6,20,31^. In a first step of the analysis of the neurophysiological signals, cluster-based permutation testing revealed modulations due to event segmentation in all three frequency bands of interest, i.e., theta, alpha, and beta frequency bands. Thus, beamforming for time intervals around responses was conducted for all three frequency bands. The largest neurophysiological activity around event boundaries in frequency bands associated with event segmentation^20^ included theta band activity in the insular cortex, alpha band activity in the insular cortex and the lingual gyrus, and beta band activity in the lingual and cingulate gyri. It is unlikely that these brain regions reflect purely motor-related activity, as the time interval examined extends well beyond the duration of pre-motor and motor processes at the neurophysiological level^20^. Additionally, the direction of effects, particularly for beta band activity, contradicts the notion that the observed effects result from post-movement rebound^65^. Furthermore, the areas identified through beamforming analysis, such as the insula and the lingual gyrus, are not typically associated with motor processes. Moreover, in right-handed individuals pressing a key with their right index finger, one would expect distinctly left-lateralized motor activity, which is absent in motor-related regions such as the SMA. The insular cortex, known as an “integration hub” for sensory inputs and involved in decision-making^66^, was associated with theta band activity which has been related to error detection driving the updating of the working event model^20^. This suggests that theta band activity in the insular cortex reflects the end of the decision process to set a boundary and update the working event model, enhancing the monitoring of sensory input for relevant information. Moreover, high alpha band activity in the insular cortex likely indicates the integration of information from event schemata into the working event model, as alpha band activity is linked to episodic memory retrieval and active working memory^20,67,68^. Similarly, high alpha band activity was found in the lingual gyrus, which plays a role in the retrieval of visual and episodic memories such as event schemata^69–71^. Moreover, the lingual gyrus exhibited high beta band activity, related to updating the working event model^20^. As the lingual gyrus is further involved in manipulating visual information over short time periods such as an event segment^72^, this suggests that beta band activity in the lingual gyrus reflects creating a new working event model based on visual inputs. Lastly, high beta band activity in the cingulate gyrus also likely reflects updating the working event model^6,20,73^. While the spatial resolution of EEG data introduces some uncertainty in localizing effects^74–76^, beamforming techniques have demonstrated reliable and robust results in setups similar to the present study^77,78^. Further analyses were conducted to elucidate the strength of these activities’ relevance to the behavioral outcomes.

Intriguingly, neither theta nor alpha band activity in the insula and the lingual gyrus influenced the dependence of event segmentation behavior on situational changes. Similarly, beta band activity in the lingual gyrus did not significantly influence event segmentation behavior. However, beta band activity in the cingulate gyrus showed a trend towards a significant association with responsiveness to situational changes: higher beta band activity in this region was associated with an increased likelihood of setting an event boundary as situational changes increased. This suggests that updating the working event model might be relevant to event segmentation behavior in adolescents. However, as only a trend toward significance remained after correction for multiple testing, this finding should be interpreted cautiously. Notably, the influence of the activity in all frequency bands and associated brain regions was not dependent on age.

Given the apparent higher relevance of the beta frequency band to event segmentation behavior in adolescence, further analyses examined the significance of the connectivity between these regions to event segmentation behavior. Both linear and nonlinear connectivity showed equal strength in both directions between the lingual gyrus and the cingulate gyrus, indicating that neither region exerts greater influence over the other. Of note, none of the connectivity measures demonstrated a direct effect on event segmentation behavior. However, when age was included as a moderator, it was revealed that the nonlinear connectivity from the cingulate to the lingual cortex influenced responsiveness to situational changes in older adolescents (according to the Johnson-Newman output from the age of 14.4 years). Notably, higher nonlinear connectivity was associated with reduced responsiveness to situational changes. This suggests that strong information flow from regions associated with higher cognitive updating functions towards visual areas such as the lingual gyrus may be detrimental to effectively considering situational changes during event segmentation. Thus, strong top-down influences between brain regions associated with the maintenance and updating of the working event model seem to hamper event segmentation behavior, possibly because the inclusion of additional information from the visual system into the working event model is suppressed by strong top-down control. This likely reflects the establishment of a new working event model with less consideration of perceptual information.

Interestingly, the study by Ghorbani et al.^6^ demonstrated that adults generally exhibit a higher responsiveness to an increasing number of situational changes compared to adolescents; however, their event segmentation behavior was not related to directed connectivity in the beta frequency band. This suggests that, while both young adolescents and adults may have rather similar connectivity patterns, older adolescents show more pronounced individual differences in segmentation behavior and their corresponding neurophysiological underpinnings during the developmental transition from childhood to adulthood. More specifically, strong top-down control from the cingulate to the lingual gyrus may facilitate a pattern of event segmentation similar to younger adolescents, as the lower responsiveness towards the number of situational changes is rather seen in younger adolescents. Conversely, when older adolescents show weaker connectivity from the cingulate to the lingual gyrus along with increased responsiveness to situational changes, this may in turn already reflect a pattern of event segmentation resembling those previously reported in adult samples^6,79^. Therefore, it is possible that a weak directed connectivity from the cingulate to the lingual gyrus represents a target state of the development of the neurophysiological system underlying event segmentation behavior. Another possibility is that this weak directed connectivity is a compensatory mechanism in advanced adolescence, perhaps strengthening the inclusion of perceptual input into the updated working event model. In any case, it seems to be important for event segmentation behavior as reported for adult samples that, besides information from memory (i.e., event schemata), perceptual input from visual regions is also appropriately considered. However, this interpretation is limited by the cross-sectional design of the study and the restriction of the age range to adolescents.

Future studies could expand beyond the region-of-interest-based connectivity used in the current study to whole-brain connectivity approaches at both the sensor level^80,81^ and the source level^53^. The latter, in particular, would allow for the analysis of connectivity not only between the most activated regions but also across the entire brain, helping to identify the most interconnected areas. This approach could provide deeper insights into key neural connections underlying event segmentation and how these connections are affected by developmental changes. Moreover, future studies should seek to overcome the current limitation that, although it is possible to delineate nonlinear connectivity, it is not yet clear how this nonlinearity is specified, which limits the interpretation of the findings. Although simulation studies with known ground truth have demonstrated robust recovery performance of the nCREANN algorithm, future work should include standardized benchmarking against a broader range of contemporary nonlinear effective connectivity estimators to further establish comparative advantages. In addition, future research might use individual structural MRI scans instead of a standard head model as a basis for source localization, particularly to account for changes in white matter throughout adolescence^82,83^. Furthermore, given the complexity of the statistical design, it would be commendable to replicate the current findings in a larger sample.

In summary, lower connectivity from the cingulate cortex to the lingual gyrus in advanced adolescence is associated with a greater responsiveness to situational changes. More specifically, weak directed nonlinear connectivity from the cingulate to the lingual gyrus seems to facilitate a responsiveness to situational changes as previously reported for adult samples in older adolescents, possibly because it improves the balance of considering input from memory (i.e., event schemata), and perceptual visual input from the environment when forming a new working event model. This pattern across adolescence suggests that as the brain matures, the balanced consideration of both sensory inputs and memories and established episodic traces becomes increasingly relevant in the context of event segmentation. These findings thus enhance the understanding of the neurophysiological underpinnings of event segmentation across adolescence and further highlight the importance of considering age-related changes in the relationship between neurophysiological activity and behavior.

## Supplementary Information

Below is the link to the electronic supplementary material.

Supplementary Material 1

## Data Availability

Raw data and code used for the described analyses are available in OSF https://osf.io/4q7sc/?view_only=df351aa23e6649fbaa4bfdb138a1182a.
