# Supplementary material for: How the influence of cingulate-lingual interactions on event segmentation changes from early to late adolescence
Source: Sci Rep. 2026 Apr 2;16:11377. doi: 10.1038/s41598-026-46182-w (PMC13049087; doi:10.1038/s41598-026-46182-w)
Supplement: Supplementary file 1 — Supplementary Material 1 [file 41598_2026_46182_MOESM1_ESM.docx]

**Supplemental Material**

**The Development of Neural Networks: How Cingulate-Lingual Interactions Influence Event Segmentation from Early to Late Adolescence**

Astrid Prochnow, Xianzhen Zhou, Foroogh Ghorbani, Veit Roessner, Bernhard Hommel, Christian Beste

**Supplemental Analysis 1: Beamforming of contrasts between Boundary Intervals and No-Boundary Intervals**

*Methods*

The segmented data including the virtual markers (see 2.4) were used for the subsequent analysis. The sources of the difference between Boundary Intervals (BI) and No-Boundary Intervals (NBI) were estimated for the theta (4-7 Hz), alpha (8-12 Hz) and beta (15-30 Hz) frequency bands using dynamic imaging of coherent sources (DICS; Gross et al., 2001) beamforming as implemented in FieldTrip (Oostenveld et al., 2010). The analysis focused on a time window from -.5 to .5 seconds relative to the response/virtual marker. The head model was based on a boundary element method (BEM). A leadfield matrix was computed with a spatial resolution of .5 cm using a rank-reduced forward model. For each subject and condition, time-frequency decomposition was performed using a multitaper method with a Hanning taper. Cross-spectral density matrices were computed for each frequency band, both for individual conditions and combined datasets. Beamforming was conducted using a DICS approach, with a common spatial filter applied to all conditions. The regularization parameter for source estimation was set to 5% to control for noise. Condition-wise source activity was estimated, and relative power differences between conditions were computed by normalizing the difference of BI minus NBI by their sum.

Grand-averaged source-difference data were thresholded at the top or bottom 1% of voxel values, depending on the expected direction of the effect obtained from the sensor-level cluster-based permutation testing (see 3.2). Only clusters containing at least five contiguous voxels were considered. To assign anatomical labels to active sources, an Automated Anatomical Labeling (AAL; Tzourio-Mazoyer et al., 2002) atlas was interpolated onto the source space. Thresholded data were then clustered using Density-Based Spatial Clustering of Applications with Noise (DBSCAN; Ester et al., 1996) with a neighborhood search radius of 1.5 times the grid size to ensure the inclusion of edge-connected voxels. The resulting cluster labels were mapped back onto the source space.

*Results*

The results are displayed in Suppl. Fig. 1. The modulation in the theta frequency band, i.e., the lower theta band activity in BI compared to NBI, was associated with the postcentral and the precentral gyri as well as the middle occipital cortex. The modulation in the alpha frequency band, i.e., the lower alpha band activity in BI compared to NBI, was associated with the calcarine and lingual gyrus, the middle and superior occipital cortex, the precuneus and the superior parietal cortex, the supramarginal and superior temporal cortex, and the fusiform gyrus. The modulation in the beta frequency band, i.e., the lower beta band activity in BI compared to NBI, was associated with the postcentral and the precentral gyri and the inferior parietal cortex.


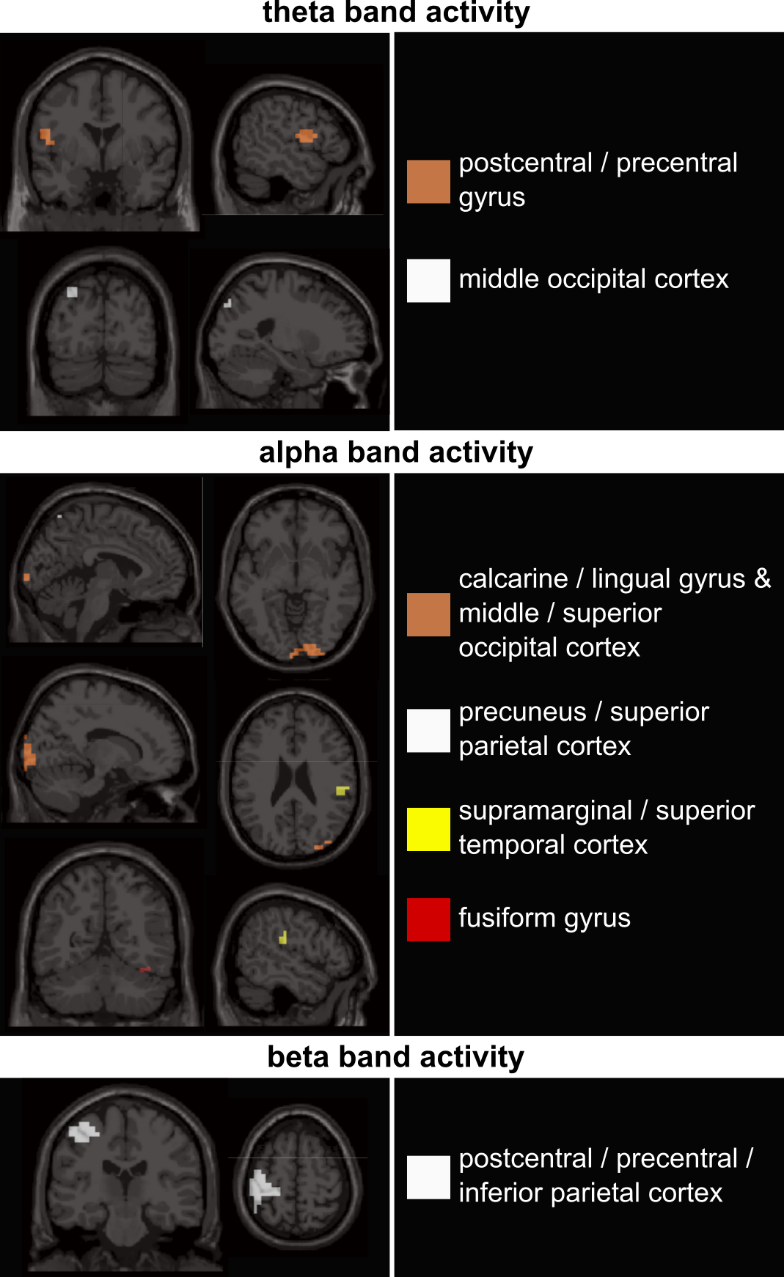


*Supplemental Figure 1.*

Illustration of the brain regions associated with the modulations in the theta (top), alpha (middle) and beta (bottom) frequency bands.

**References**

Ester, M., Kriegel, H.-P., Sander, J., Xu, X., 1996. A Density-Based Algorithm for Discovering Clusters in Large Spatial Databases with Noise. kdd 96, 226–231.

Gross, J., Kujala, J., Hamalainen, M., Timmermann, L., Schnitzler, A., Salmelin, R., 2001. Dynamic imaging of coherent sources: Studying neural interactions in the human brain. Proc. Natl. Acad. Sci. U.S.A. 98, 694–699. https://doi.org/10.1073/pnas.98.2.694

Oostenveld, R., Fries, P., Maris, E., Schoffelen, J.-M., 2010. FieldTrip: Open Source Software for Advanced Analysis of MEG, EEG, and Invasive Electrophysiological Data. Computational Intelligence and Neuroscience 2011, e156869. https://doi.org/10.1155/2011/156869

Tzourio-Mazoyer, N., Landeau, B., Papathanassiou, D., Crivello, F., Etard, O., Delcroix, N., Mazoyer, B., Joliot, M., 2002. Automated anatomical labeling of activations in SPM using a macroscopic anatomical parcellation of the MNI MRI single-subject brain. Neuroimage 15, 273–289. https://doi.org/10.1006/nimg.2001.0978
